# Knowledge, Attitude and Practice of Main Stakeholders towards Human Papilloma Virus Infection and Vaccination in Mombasa and Tana-River Counties in Kenya: A Qualitative Study

**DOI:** 10.3390/vaccines9101099

**Published:** 2021-09-28

**Authors:** Diana Wangeshi Njuguna, Nour Mahrouseh, Osarugue Victory Isowamwen, Orsolya Varga

**Affiliations:** 1 Department of Public Health and Epidemiology, Faculty of Medicine, University of Debrecen, 4032 Debrecen, Hungary; diana.njuguna@med.unideb.hu (D.W.N.); nour.mahrouseh@med.unideb.hu (N.M.); 2 Doctoral School of Health Sciences, University of Debrecen, 4032 Debrecen, Hungary; 3 Faculty of Medicine, University of Debrecen, 4032 Debrecen, Hungary; Isowamwen@mailbox.unideb.hu; 4 Office for Supported Research Groups, Eötvös Loránd Research Network, 1052 Budapest, Hungary

**Keywords:** HPV vaccination, cervical cancer, HPV vaccine, national immunization program, vaccine hesitancy

## Abstract

Following a successful Human Papilloma Virus (HPV) vaccination pilot in 2013–2015 in Kitui county, Kenya introduced the HPV vaccine in October 2019 with a goal to immunize approximately 800,000 girls annually against HPV. Our study assessed the knowledge, attitudes, and practice of affected groups towards HPV infection and vaccination in two counties of Kenya. Semi-structured interviews from children aged between nine and thirteen years and key informants comprising of parents, head teachers, community leaders and health workers involved in HPV vaccination in health facilities from Mombasa and Tana-River counties were conducted. Content was analyzed thematically and coded for emerging themes using the QRS Nvivo 12 Plus (QRS International, Doncaster, Australia) software package. From our findings, a significant proportion of participants, especially children, have limited knowledge of the subject. Vaccination of boys was opposed by most participants. Parents and the community members are not in favor of HPV vaccination, as compared to the other groups. A similar pattern of inadequate knowledge and strongly opposed attitudes was observed in Tana-River and Mombasa. Active community involvement in primary prevention strategies may promote the uptake of the vaccine which can be achieved by robust awareness, modifying the negative beliefs about HPV vaccine and encouraging the perceptibility of HPV vaccination.

## 1. Introduction

Human Papilloma Viruses (HPVs) are a group of sexually transmitted viruses that are implicated to cause cervical cancer [1]. Evidence from the World Health Organization (WHO) reveals that HPV is responsible for 70% of cervical cancers and precancerous cervical lesions [2]. Globally, the most prevalent HPV oncogenic types are HPV 16 and HPV 18. Persistent untreated infections with these high risk types lead to the formation of abnormal cervical precancerous cells [3,4]. Evidence shows that HPV vaccination reduces the incidence of cervical cancer by providing prophylactic efficacy against cervical pre-cancer in adolescent girls and women who received the HPV vaccination [1,5]. It is recommended that girls between the age of 9–14 be vaccinated since they are not sexually active (hence no prior exposure to HPV) [6]. However, gender-neutral vaccination is recommended in several countries [7]. The effectiveness and benefits of the HPV vaccine have been studied in several countries as this issue has been treated as a public health priority [8,9,10]. Robust evidence of a reduction in cervical cancer incidence exists in developed countries such as Australia, the United Kingdom, and the United States, where vaccination coverage is high [8,11,12,13].

Modelling projections suggest that high HPV vaccination coverage among girls in low-and middle-income countries (LMICs) can lead to cervical cancer elimination. Thus, the introduction of HPV vaccination is of especially urgent need in developing countries where there is a high burden of cervical cancer [5,14,15,16,17,18,19]. A study on the trends of cervical cancer in Sub-Saharan Africa acclaimed that prophylactic vaccination for HPV and mass screening for cervical cancer are the key prevention strategies for cervical cancer [9]. Comprehensive HPV vaccination and public education could significantly prevent over half of all cervical cancer deaths [10].

Globally, the burden of cervical cancer is greatest in developing countries, including the Eastern Africa countries. In terms of cancer incidence in Kenya, cervical cancer is second only to breast cancer, with 5250 (11%) new cases per year in both men and women [20]. In Kenya, the most prevalent oncogenic types among women are HPV 16, followed by HPV 18. The burden of cervical HPV infection prevalence (%) includes women with normal cytology (9.1%), low-grade cervical lesions (LSIL/CIN-1) (21.4%), high-grade cervical lesions (HSIL/CIN-2/CIN-3/CIS) (45%) and cervical cancer (63.1%) [21]. The low number of binding and old policies, poor implementation of cancer control policies in Sub-Saharan Africa, poor morbidity and mortality records, and lack of cancer registries mean that the long-term problem of cervical cancer is likely to persist for a long time in these countries [22,23,24].

Globally, HPV vaccines were rolled out in 2006 with many countries showing a tremendous scale up by adopting the HPV vaccine in their national immunization schedules [25,26]. Following a successful HPV vaccination pilot program in 2013–2015 in Kitui county [27], Kenya introduced the HPV vaccine in October 2019 with a goal to immunize approximately 800,000 girls annually against HPV per year [28]. The vaccines were provided through the support by the Global Alliance for Vaccines and Immunizations and the WHO, while the United Nations Children’s Fund was to help the government of Kenya to disseminate evidence based information on the benefits, safety, and efficacy of the HPV vaccine to the community [29]. Although the Kenyan government planned to introduce the HPV vaccine to the routine immunization program, it has not been implemented yet. There are two types of HPV vaccines recommended by the WHO: Cervarix and Gardasil. Cervarix is a bivalent vaccine containing virus like particles (VLPs) of HPV 16 and 18 (which are the high-risk HPV types) while Gardasil targets both HPV 16 and 18 in addition to VLPs of HPV 6 and 11 which are implicated for 90% of external genital warts in both men and women [30,31].

Studies in routine vaccination with three doses of HPV vaccine have been recommended for adolescent girls and boys at 11 or 12 years of age. However, vaccine uptake remains suboptimal globally. This has been associated with low levels of vaccine awareness [32,33,34]. There are many factors that lead to poor compliance to the HPV vaccination program which in turn highly contribute to the failure of vaccine uptake. These factors include unreasonable parental fears, personal, cultural and religious reasons, and parental misinformation among other factors [35,36,37]. Stakeholders’ acceptance determines the compliance to the vaccination exercise and greatly contributes to its success [38]. Health care providers and all stakeholders affected by the introduction of HPV vaccination need to address the concerns of parents and children in order to ensure effective HPV vaccination [39].

Generally, the perception of the communities in Kenya in regards to HPV vaccination revolve around perceived mistrust towards new vaccinations, insufficient knowledge about HPV infections and HPV vaccination, and high levels of misinformation due to inadequate top down training of stakeholders [36,37,38,40]. A study that sought to compare vaccine acceptability and knowledge in Eldoret, Kenya highlighted the need to increase exposure of the HPV vaccine which in turn could improve knowledge on cervical cancer, HPV infection, HPV vaccination, and its prevention strategies [37]. Elsewhere in Kitui, Kenya, a study among school teachers revealed that despite low levels of knowledge about HPV vaccine and cervical cancer, there was high acceptance of the vaccine, despite barriers such as inaccessibility to the school, girls’ absenteeism, and fear of side effects [36]. In western Kenya, a study on practices, attitudes, and knowledge among mothers of adolescent girls indicated that there was a positive attitude and desire towards the use of HPV vaccination despite the evident low levels of knowledge [41]. Undoubtedly, having the basic knowledge on the natural history of cervical cancer is a great milestone to its prevention and control [42].

Our understanding of the views on HPV vaccination in Kenya is fragmented, little is known about HPV vaccine uptake in different parts of the country, and moreover, the attitudes of stakeholders towards vaccinating boys are not known at all. In this regard, our research work seeks to bring an insight on the status of the HPV vaccination program and perceptions about the introduction of HPV vaccination to the 10 to 11-year-old boys and girls in the coastal region of Kenya (i.e., Mombasa and Tana River counties).

The objectives of this study were twofold: (1) to assess the knowledge and attitude of HPV vaccination among school boys and girls, parents and other key informants comprising of head teachers, health workers and community leaders regarding HPV vaccination, and (2) to gain information about the practice of HPV vaccination programs in Mombasa and Tana-River counties in Kenya and potential improvements.

## 2. Materials and Methods

### 2.1. Study Area

The interviews took place in two counties, Mombasa and Tana-River, located on the Indian Ocean coast, where no similar survey has been conducted before. The two counties are characterized by a different socio-economic situation, which allows comparison of the results from urban settings (Mombasa County) and rural settings (Tana-River). Mombasa county has one of the highest Per Capita Gross County Product (GCP) of 271,039 Kenya shillings in 2013–2017 [43]. The GCP is a measure of how much each county contributes to Kenya’s gross domestic product (GDP) and may therefore be interpreted as the “County GDP”. Mombasa is the second largest city after Nairobi, the capital of Kenya with a relatively high proportion of foreigners and a cosmopolitan population; it is a tourism-based city. Tana-River, by contrast, is one of the underdeveloped counties, among the ones with the lowest Per Capita GCP of 106,894 Kenya shillings in 2013–2017 [43]. The population is composed of three communities; Somali, Pokomo and Orma. The county is characterized by local conflicts and drough [43,44]. Both Mombasa and Tana-River counties are located in the Kenyan coastal region and they rely economically on natural assets from marine resources. These communities depend on the ocean for their social and cultural security [45].

### 2.2. Study Design and Sampling

In this descriptive qualitative study, individual interview participants were purposively selected using a maximum variation sampling strategy, with the inclusion criteria of having at least a boy or a girl aged between 9–13 years, parent, head teacher, community leader and health worker related to HPV vaccination in both Mombasa and Tana-River counties. The interviewees were drawn from different population categories of economic class, gender, age, place of residence and religion. From both counties, we managed to interview 7 school boys and 19 school girls aged between 10–13 years all from different schools. Among the key informants, interviews were conducted from 10 parents, 23 head teachers, and 22 community leaders who comprised of chiefs, village elders, religious leaders and community health volunteers. The 21 health workers comprising of nurses and public health workers had been involved in HPV vaccination in health facilities. After transcription of interviews participants were assigned numbers randomly to preserve their anonymity.

### 2.3. Data Collection

Data collection was conducted through semi-structured interviews of which the guide was drafted focusing on three broad themes: for participants’ knowledge related to cervical cancer, their attitude towards cervical cancer prevention and their views on possible ways to address the barriers in current practice, see the topics in the Supplementary File S1a. These major themes were targeted since they determine the effective use of the vaccine. The development of this guide was based on the WHO guideline outlining the community health workers, and primary and secondary level health care providers, as the implementers of the HPV vaccination programs [44]. The tool had related questions although they were customized for the different groups of participants in the study population. Data collection was conducted in an intimate environment of the participant or in the shaded environment near the parents or, in case of adult informants, in their offices. Boys’ and girls’ interviews were carried out in the schools in a private secluded part of the classroom. All of the semi-structured interviews were undertaken by one member of the research team (DN) who has significant experience in qualitative research. The interviews were recorded using a digital voice recorder and notes were taken. Data collection was conducted between September 2020 and February 2021. The language of the interviews was either English or Swahili, according to the participant’s preference. The average length of the interview was 20 min.

### 2.4. Data Analysis

The audio files were transcribed verbatim. The original list of codes was based on research questions and literature review. Content was analyzed thematically, indexed, and coded inductively for emerging similar themes using the QRS Nvivo 12 Plus software package (QRS International, Doncaster, Australia) for content analysis of unstructured qualitative data. The initial open codes were sorted into sub-themes based on their similarity. These subthemes were clustered and refined to form broad themes and debated in the research group. The knowledge nodes responses, based on current evidenced based knowledge, were categorized into three according to the accuracy of the response as correct, lack of an answer or severe misunderstanding. Under attitude nodes, the opinion of others was classified depending on the nature of the attitude as supportive, opposing or neutral. Data collection, analysis, and reporting followed the Standards for Reporting Qualitative Research (SPQR) guideline see (Supplementary File S1b) [46].

### 2.5. Techniques to Enhance Trustworthiness

We ensured consistency during protocol preparation, data collection, development of a coding system, interrater reliability, and data analysis by following Guba and Lincoln’s criteria for determining rigor in qualitative research [46,47]. Two interviews per group were coded by two authors (DN, OV), for which the degree of similarity was determined by calculating the interrater reliability using the Nvivo software (QRS International, Doncaster, Australia). DN coded all the interviews and OV coded a sample (10%) of the interviews. The randomly selected sample (*n* = 10) was structured to include a similar proportion of interviews from each county and each stakeholder group. Once satisfactory reliability was established, DN then proceeded to code the remaining data independently [48]. Cohen proposed that Kappa scores should be interpreted using the following: values ≤0 indicate lack of agreement, values 0.01–0.20 indicate no or little agreement, values 0.21–0.40 indicate fair agreement, values 0.41–0.60 indicate moderate agreement, values 0.61–0.80 indicate substantial agreement, and values 0.81–1.00 indicate nearly perfect agreement [47]. Kappa scores per theme were: knowledge 0.79, attitude 0.83, and practice 0.75.

### 2.6. Ethical Issues Pertaining Human Subjects

Permission to conduct the research was granted by the University of Eastern Africa, Baraton (UEAB) Research and Ethics Board in Kenya and a research permit from National Commission for Science, Technology and Innovation (NACOSTI) License number NACOSTI/P/20/6514. Written informed consent was granted by all the adult participants prior to the interview and on behalf of the minors, the parents and teachers signed the informed consent, see Supplementary File S1c.

## 3. Results

### 3.1. Demographic Characteristics of Participants

Women dominated our sample, with 53 of the 76 adults interviewed being women. Among the 10 parents interviewed 9 were female, additionally, in other adults’ groups like the nurses in the health worker’s group consisted of females, exclusively. The age of parents, health workers and nurses ranged from 30–45 years, while the age of community health workers ranged from 35–60 years. The boys and girls ages ranged between 10 and 13 years.

### 3.2. Burden of Cervical Cancer

The burden of cervical cancer was assessed among the health workers and community leaders. The responses were consistent and emphasized that late diagnosis was rampant and the rate of mortality from cervical cancer is quite high. As participant 32, a health worker noted

*“...Yes, cervical cancer is a real problem in my community. We have had cases of several women who get diagnosed at very late stages and end up losing their life and practically we lost one of the community health volunteers to cervical cancer because it was diagnosed at a very late stage”.*

Additionally, an upsurge in the frequency of positives tests during cervical cancer screening was reported by participant 57

*“...Yeah it is, well of late we are getting cases because we have been encouraging people to do cervical cancer screening, and I think we got some few cases, so cervical cancer it is real in our community”.*

The burden of disease was not only observed by health workers, for example participant 19, a head teacher said that,

*“...Yes, it is rampant. So many people die with cervical cancer”.*

Participant 35, a community leader expressed that,

*“...It is a very serious problem, because, in the past, we did not understand the disease, but this time, we have seen women; it has become very challenging, because many are suffering from the disease. Before it is known, the mother loses her life, she dies“.*

### 3.3. Knowledge on Cervical Cancer, HPV and HPV Vaccination

The knowledge theme had seven nodes comprising of answers related to knowledge about HPV, goal of HPV vaccination, prevention of cervical cancer and HPV, risk of catching HPV or cervical cancer, what is cervical cancer, what is the cervix, and what is the goal of Pap smear. The answers were classified according to their correctness (i.e., correct, lack of an answer, and an answer with severe misunderstanding).

**Figure 1:**
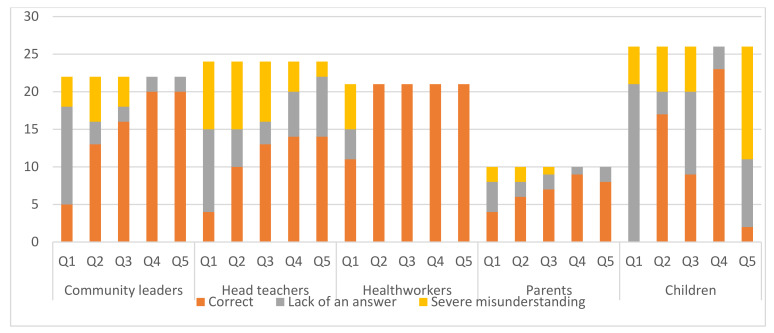
Knowledge on Human Papilloma Viruses (HPVs) infections, HPV vaccination and cervical cancer. The figure shows the number of participants in each category (*y*-axis) and their responses (correct in orange, lack of an answer in grey and severe misunderstanding in yellow) to the questions asked to assess their knowledge. Q1, Question 1: What is HPV; Q2, Question 2: Goal of HPV vaccination; Q3, Question 3: Prevention of Cervical cancer and HPV; Q4, Question4: Risks of catching HPV; Q5, Question 5: What is cervical cancer.

Most responses by healthcare workers were correct while the highest percentage of incorrect or no responses were by children. The lack of appropriate biological knowledge was particularly noticeable for children (see Figure 1). The responses that had high percentage of correct responses from the groups combined were from the questions on risks of catching HPV and cervical cancer, what is cervical cancer, prevention of HPV and cervical cancer, and goal of HPV vaccination.

Considerable numbers of responses with serious misunderstanding were found, such as 37% of head teachers when asked what HPV is, 26% of community leaders when asked what the HPV vaccine is for, and 57% of children when asked what cervical cancer is. This is echoed by the quote by participant 23, a head teacher on the importance of HPV vaccination,

*“…I don’t think it’s important at this point because I have never heard of a young child having the cervical cancer or something”.*

Another participant 40 who was a parent felt that,

*“…if she received this vaccine, even if she goes to get married, if she meets the man who is infected with the HPV virus, they do not get it. She will be safe”.*

The responses on how to prevent HPV and cervical cancer were acceptable in most groups, with the health workers and community leaders having 99% and 74%, respectively. However, others demonstrated no or limited knowledge on the prevention of cervical cancer and HPV. Participant 11, a community leader said,

*“…I can’t tell the prevention because I don’t know what is cervical cancer”.*

A parent had the feeling that,

*“…Preventing will be by giving medicine, try to prevent it by giving medicine that will prevent it from happening”.*

Severe misunderstanding was displayed on the prevention of HPV and cervical cancer by participant 1, a community leader,

*“…prevention mmmmmh mainly this disease we can prevent by your lifestyle maybe. The food you eat, that is your lifestyle”.*

While the answers to the question on knowledge of the risk of HPV and cervical cancer were adequate in all groups, many participants had difficulty in answering the question on what HPV is. Some said they had only heard about human immunodeficiency virus (HIV) and not HPV; participant 31, a health worker uttered,

*“…All I know is the one that is associated with HIV”.*

An answer by participant 23 showed a severe misunderstanding,

*“… acidic food actually, kind of thinking is a bit, delays it to the feelings of papilloma because once you eat this thing you feel some changes and then you relate it to papilloma”.*

Most adult participants gave a correct answer to the question what is cervical cancer. Some were shy and chose to stay silent and not respond, especially in the children’s (78%) and head teachers’ (19%) group. A boy said that,

*“…I have heard it is a terrible disease that is killing people”.*

Participant 56, a head teacher said,

*“…Yeah, you know I don’t have knowledge of health, otherwise I have heard about it”.*

The level of knowledge about the purpose of the Pap smear was moderate among the three groups asked, with parents (66%), head teachers (63%) and community leaders (30%) having a reasonable level of knowledge and giving a correct answer. This question on cervix was asked to all the groups except the health workers. A considerable portion of the community leaders (61%) gave a correct response then the head teachers (43%), parents (36%) and the children (20%). The children’s group responded most silently (78%).

### 3.4. Attitudes and Views Regarding HPV Vaccination

The attitude theme had six nodes which were access to information, efficacy, safety, opinion of others, cultural beliefs, and religious beliefs. With the exception of the opinion of others, these child nodes were linked to the attitudes and perceptions of individual participants. The opinion of others meant how the interviewee thought other people (children, parents, class teachers, health workers and community leaders) felt about HPV vaccination. The opinions of others were categorized depending on the nature of the attitude (i.e., supportive, opposing, and neutral).

In regards to acceptance of the HPV vaccination, perception, and willingness to participate in the HPV vaccination exercise, the participants noted that challenging access to and inadequate availability of information as a factor influencing attitude. Participant 42, a community leader reported that the community lacked information,

*“…they still need to be educated because the information they have is still low”.*

Low literacy (*n* = 11) was also mentioned as a barrier to the uptake of the HPV vaccine.

Only a handful of the participants alluded to the efficacy of the HPV vaccine. A few did not have confidence in its ability to prevent cervical cancer (*n* = 13). A parent had this to say,

*“…am not quite sure if it can work, because I have heard many controversies about it. There have been uncertainties of whether it can actually work”.*

Quite a number of participants (*n* = 72) believed that the HPV vaccine could be ineffective in boys because they do not have a cervix. This view is depicted in a quote by participant 20, a head teacher,

*“…it doesn’t relate to the boys or do they also have ovaries”.*

Participant 2 a community leader felt that,

*“…it something that they cannot get because maybe they don’t have a uterus”.*

Safety concerns were the most significant determinant of attitudes, with the highest number of references to this. There was a uniform pattern observed in both counties with birth control misconceptions having high percentages among the participants. From the Table 1 below, Tana-River County groups as compared to Mombasa county groups had higher percentages in the birth control misconceptions. Children in both counties were most likely to mention acute side effects, while parents were afraid of the unknown.

Some of these views are illustrated. Participant 6, a community leader referring to the community, stated that,

*“…they rejected and sent false messages that the vaccines are for family planning and it would affect their children in many ways”.*

As most other participants alleged that the HPV vaccine is associated with birth control, a girl expressed this,

*“…yes, some said they do not want to be injected. Others said, the vaccine spoils the womb of girls”.*

The fear of unknown (*n* = 33) was mentioned frequently by the participants. Participant 31, a health worker, articulated their fears,

*“…so far I myself also, have fear of unknown. What if you’re taking it as a prevention then eventually you come into contact with cervical cancer as a result of you being vaccinated and then you get cervical cancer then what would be the repercussion of this vaccine, Is it 100 effective?”.*

Among the adult groups, some even expressed that they had more fear than the children themselves, others cited the fear was due to the newness of the vaccine and the negative beliefs from other community members about the HPV vaccine. A parent apprehensively stated this,

*“…Yes. I have fear because, as a parent, I am at risk of getting the cancer, and my baby is also at risk of getting this cancer”.*

Most of the girls had fear of the injection. This is demonstrated by the following quote from a girl,

*“…Some said they were scared. Others when their turn to be injected came, they moved backwards”.*

The actual side effects were mentioned severally though none of the participants could recall whether they observed any actual side effects after the vaccination. The following were the responses to the question if they had encountered or heard of any common or severe side-effects from HPV vaccination. Participant 32, a health worker re-counted this,

*“…From the girls who have received the jabs and come for the follow-up vaccination we’ve not yet seen any side effect”.*

When interviewees talked about the attitudes of others, cultural and religious beliefs with consistent impact on attitude in opposing the HPV vaccination program in both counties were emphasized. Most of the participants mentioned that the local culture was an impediment to HPV vaccination. For example, participant 37, a community leader said that,

*“…in our community, culture, that is, is the biggest factor that has hindered. Because, they believe, the HPV vaccine, is to limit children, child bearing, that is, as in a child when she is older, they give her the vaccine so that, she does not have more children in the future”.*

Other participants strongly believed in their faith hence opposing the vaccination. This was demonstrated by participant 46, a head teacher:

*“…Religious reasons. Some have faith say that no medication to them should be administered right from birth, they should just live like that and only Jesus Christ would save them. Such faith becomes really a challenge to us”.*

Participant 58, a community leader acknowledged that,

*“…you know mostly the people who are living in this locality are Muslims, family planning is prohibited in the holy Quran that is their perception”.*

Additional irrational statement about beliefs in their community was given by a parent on the goal HPV vaccination that,

*“…They also say that you are being injected so that you join Illuminati”.*

Many respondents found the attitude of the community is rather opposing, when responding to reasons why some girls did not complete the doses, participant 52, a head teacher speculated that,

*“…maybe after getting the first dose, maybe a parent was misled by someone maybe in the community that don’t repeat this”.*

Table 2 below illustrates that interviewees think that a higher proportion of community members and parents are opposed to HPV vaccination in both counties. The teachers and community leaders on the other hand supported, followed by the health workers, children and the media.

### 3.5. Practical Recommendations to Improve HPV Vaccination Compliance

The theme about practice and recommendations was categorized into seven nodes. This covered consent process, COVID pandemic, inclusion into the national immunization schedule, pre-vaccination preparedness, building capacity for health workers and community leaders, community involvement and sensitization.

During the informed consent process, 37 interviews demonstrated that children, health workers and parents were involved in making informed decisions about HPV vaccination. Interviews showed that the girls were informed and their parents were part of the consent process. Participant 12, a head teacher told us that,

*“…the students were called and they were told to bring their parents and their parents were talked to and they were given consent forms to sign which there are others that refused.”*

The health workers reported that the COVID pandemic had an effect on the continuation of the HPV vaccination exercise following abrupt closure of schools. Participant 29, a health worker voiced that,

*“…Before the COVID we had already arranged to go to certain schools so that we can give them there.”*

This directly led to halting of all the planned vaccination activities by the county hospitals in collaboration with the schools. The children who received first doses experienced delay of the consequent dose. This is captured in this quote by participant 66, a health worker,

*“…the COVID pandemic drew us back again because when we were in top gear trying to do dissemination of information that’s when lockdowns came in, people are basically afraid to come to the hospitals”.*

Majority of the participants gave recommendations on sensitization. A parent expressed that,

*“…it would be good for the community to be sensitized so that they know the importance of the vaccine”.*

Since the HPV vaccine is yet to be added to the national immunization schedule, a few participants felt that its inclusion will make it compulsory for all the eligible children. A quote from participant 63 a health worker endorsed this,

*“…the government make it compulsory for every child to receive this vaccination. So if after policy is in place it will be everybody abide by it to make sure that the child is given this vaccination”.*

Some respondents argued that there is a need for thorough pre-vaccination education, coupled with community involvement and capacity building of health workers and community leaders. Participant 32, a health worker had this to say,

*“…We will also like to involve the politicians, the spiritual leaders because they are the people who influence most of the peoples’ decisions, so if we involve them at least to create a positive impact when it comes to the vaccination and also if the ministry of education works together with the ministry of health to ensure that each and every girl gets the jab; at least no girl is left behind”.*

## 4. Discussion

### 4.1. Summary of Major Findings

A significant proportion of adults have limited knowledge of the subject; health workers were the most informed. Despite the fact that children are also involved in decisions about vaccination, children have very limited knowledge about HPV infection, HPV vaccination, and cervical cancer. Broad irrational fears and misinformation about HPV vaccination exist in society. The vaccination of boys is not supported by the majority of participants. Parents and the community members in general are not in favor of HPV vaccination, while teachers, health workers, and community leaders are. A similar pattern of inadequate knowledge and strongly opposed attitudes was observed in Tana-River and Mombasa.

### 4.2. Integration with Prior Work, Implications, Transferability and Contribution to the Field

Most of the participants indicated that they had heard about either cervical cancer or HPV but they could not remember what it was, and many had barely heard about a Pap smear. In terms of prevention, a lot of participants were aware of the HPV vaccine as a preventive measure. Considerable number of participants were cognizant that they were at risk of catching HPV infection and cervical cancer, especially due to early onset of sexual activity and having multiple sexual partners (this is in agreement with Yamaguchi) [48]. This corresponds to a study on cervical cancer screening and acceptability of HPV vaccine among women in Kilimanjaro [49]. Although great support has been established since HPV vaccines have been available since 2006/2007 to promote and educate women globally and especially in LMICs [50], the WHO’s recommendation on the HPV vaccine is that in order to achieve the much needed high levels of HPV acceptability, there is urgent need to conduct campaigns to create awareness and provide more accessibility [6,34,51].

Although knowledge gaps among health workers were evident, our findings proved the availability of information, to some extent. Information available in the community is derived mainly from seminars and workshops arranged by health authorities. Apparently, the information given to girls and parents prior to vaccination was suboptimal [52]. Thus, adequate training and other information channels are recommended for successful implementation of the vaccination exercise. A meta-analysis on educational training of health providers in the United States of America has found that increased training and communication of health workers can improve the efficacy of vaccination campaigns [53].

Misinformation about HPV vaccination is common among community members and has influenced attitudes towards HPV vaccination uptake. A majority of the participants insinuated that the HPV vaccine was a birth control method and it is alleged that it is laced with family planning components to sterilize the girls. Others even went to the extent to report that the HPV vaccine itself was an introduction of cervical cancer to the girls. This was further complicated by the fact that some religious leaders were sources of these statements as these arguments were already reported by Otieno et al. [54,55]. Misinformation causes considerable damage, and it is now incumbent on health workers to get the right HPV vaccination messages out to the masses [56].

A variety of reasons were given by participants for not vaccinating children in their communities, including irrational fear of unknown vaccines, deviation from cultural and traditional norms, pressure from community members, husbands barring their wives from vaccinating the girls, and absurd beliefs such as vaccination being an initiation into the Illuminati. Findings from our study confirm that cultural norms and religious factors have shaped the HPV vaccination landscape [50,57]. Most of the parents had the desire to have their daughters vaccinated so long as the vaccination was to prevent the girls from getting cervical cancer later in life [52]. These effects can be mitigated by proper information [58], such as the success rate of promotion campaign targeting the community and leaders, in well bounded community as the Kenyan one, the community can be led by the village elders who will take up this role [53].

Not unexpectedly, safety concerns have been raised about the effectiveness of the HPV vaccine. Some reported that they had fear of the side effects or their children dying, the girls also expressed fear of being injected. However, there is no evidence of association of HPV vaccine and adverse effects [59] and HPV vaccine is safe and has been shown to have little or no adverse events [60].

There were disparate responses to the question on whether to vaccinate boys or not. One of the boys expressed a desire to also be immunized and this agrees with Grandahl et al. findings on “I also want to be vaccinated!” where the boys were in favor for the HPV vaccine [57]. Most participants were against vaccinating the boys because they either didn’t have a cervix or cervical cancer affects women and not men, hence there was no need to vaccinate the boys. This depicted a lack of awareness about the diseases caused by HPV infections which finding is consistent with a study investigating HPV vaccination in boys [61]. Other participants felt that the priority should be given to girls then later the program can be expanded to include the boys. These kinds of remarks correspond to a study that compared HPV knowledge and intentions among parents of boys and girls where the parents felt that it was more important to vaccinate girls than boys [62]. The responses were opposed and some participants questioned if boys had a cervix. Many associated the HPV vaccine being for girls due to the prevention of cervical cancer, exclusively. This is a common perception in the community as revealed in literature [7,62,63].

The opposing attitude from the community and parents was quite conspicuous from both counties. The disapproval has been observed in other studies where community groups and parents have had controversy and opposed the HPV vaccination due to their irrational fears, concerns, and arguments. Active involvement of the health workers in dialogues to address the parents’ and community’s concerns is a definite way to mitigate this [42,63]. Capacity building of health workers and community leaders is essential as these are the drivers of disseminating the information to the community.

The parents and children were involved in the consent process. From our findings, the adolescent girls exercised high level of autonomy as demonstrated by the nurses’ response to the question on how the adolescent girls felt about being vaccinated. This is in line with a study conducted in South Africa on factors influencing vaccine uptake. However, in this study, the nurses did not express any difficulty initiating these conversations about sex with the adolescents. This contradicts with our findings on the role of the health care workers on the difficulty in initiating conversations about sex with the adolescents [42].

The COVID-19 pandemic was seen to have affected the practice of HPV vaccination exercise. The safety and efficacy concerns towards the COVID-19 vaccine observed across countries and suspension of preventive activities greatly influenced the uptake of HPV vaccination [64,65]. It was mentioned that the girls either missed or had delays for the second doses. The school programs were halted due to the lockdown that saw many students stay at home. This has also been the case in several other parts of the world and health care providers have been urged to make plans to recover the HPV vaccination programs to avoid long term consequences [66].

From the health workers, it was revealed that the HPV vaccine has yet not been included in the national immunization schedule. They recommended that it should be added as this would curb the failure and forgetfulness to go for the second doses. Evidence from literature shows that countries will have to increase their national immunization budgets due to the inclusion of the HPV vaccination to the routine immunizations [67]. Community involvement and pre-vaccination preparedness was recommended, which may aid in vaccine acceptance consequently influencing the uptake of the HPV vaccination [68].

### 4.3. Implications

This study reveals the ailing obstacles to the HPV vaccination uptake from two counties in Kenya. Similar results for the two counties might be generalized to the country, as the Kenyan population is not as diverse and the majority of residents live in urban settings. The uniform recommendations from a portion of the participants, especially the health workers and community leaders on sensitization and community involvement from both counties demonstrates a willingness to promote HPV vaccination if efforts are concerted.

### 4.4. Limitation

The interviews were conducted in only two counties in Kenya, which is considered a shortcoming of our study. For our data collection, we used semi-structured, face-to-face interviews, which ensures a high degree of content validity as we collect first-hand detailed information on the subject. Selection bias is inherent in this methodology, and certainly affects this work. Selection bias can affect the interpretation of the data.

## 5. Conclusions

The study adds to the existing knowledge on the impact of low HPV vaccination uptake in Kenya. Involving the community in primary prevention strategies may promote vaccine uptake. This can be achieved by raising awareness, changing negative beliefs about the HPV vaccine, and promoting HPV vaccine perceptibility. The personal presence of experts helps to spread knowledge about cervical cancer, HPV infection, and HPV vaccination. Considering the introduction of uniform methods for tracking HPV, vaccination programs will improve HPV vaccination coverage and influence policy making. A grassroots social mobilization is needed to improve the willingness of the population to vaccinate, with accurate information for parents and teachers, who are key implementers and promoters of HPV vaccination (yet often do not support vaccination). In addition, a campaign to reach children is critical; although they cannot decide on their own, our results demonstrate that parents in Kenya take their concerns into account. Future studies should focus on mass awareness and education about HPV vaccination and cervical cancer, as well as efficacy of innovative ways and tactics to deliver messages to the community.

## Figures and Tables

**Table 1:** Safety nodes from both counties.

	Absence of Actual Side Effects	Birth Control Misconceptions	Fear of the Unknown
Parents	6% (6/10)	45% (10/10)	49% (7/10)
Health workers	16% (10/21)	51% (7/21	34% (7/21)
Head teachers	22% (20/23)	64% (14/23)	14% (5/23)
Community leaders	8% (20/22)	64% (16/22)	28% (11/22)
Children	59% (10/26)	28% (3/26)	13% (1/26)
Tana-River County	13% (35/50)	74% (26/50)	13% (6/50)
Mombasa County	14% (41/52)	50% (32/52)	36% (27/52)

The table shows how the safety nodes were distributed in the different groups and from each county.

**Table 2:** Opinions of others towards the HPV vaccination program.

	Neutral	Opposing	Supportive
Children	14% (5/36)	19% (7/36)	78% (28/36)
Community leaders	27% (6/22)	23% (5/22)	82% (18/22)
Community members	21% (14/67)	79% (53/67)	39% (26/67)
Healthcare workers	26% (10/38)	39% (15/38)	82% (31/38)
Media	0%	17% (2/12)	67% (8/12)
Parents’ opinions	12% (8/66)	71% (47/66)	52% (34/66)
Teachers	13% (3/23)	4% (1/23)	83% (19/23)

The table shows the opinions of others—thoughts that the participants randomly made about the children, community leaders, community members, healthcare workers, media, parents’ opinion and teachers—to the HPV vaccination program categorized into neutral, opposing or supportive.

## Data Availability

Data available on request due to privacy restrictions.

## Supplementary Materials

The following are available online at https://www.mdpi.com/article/10.3390/vaccines9101099/s1. File S1a Semi-structured interview guide; File S1b Standards for Reporting Qualitative Research (SRQR) checklist; File S1c Project summary and Informed consent form.
