# Supplementary material for: Knowledge, Attitude and Practice of Main Stakeholders towards Human Papilloma Virus Infection and Vaccination in Mombasa and Tana-River Counties in Kenya: A Qualitative Study
Source: Vaccines (Basel). 2021 Sep 28;9(10):1099. doi: 10.3390/vaccines9101099 (PMC8538985; doi:10.3390/vaccines9101099)
Supplement: Supplementary file 1 [file vaccines-09-01099-s001.zip › vaccines-1371793-supplementary.pdf]

Knowledge, Attitude and Practice of main stakeholders towards Human Papilloma Virus Infection and Vaccination in Mombasa and Tana-River counties in Kenya: A qualitative study

Diana Wangeshi Njuguna, Nour Mahrouseh, Osarugue Victory Isowamwen and Orsolya Varga

Supplementary S1(a): Semi-structured interview guide for community leaders, health workers, girls and boys and parents/guardians and key informants comprising of school teachers:

The guide consists of two sets of questions, the first set is prepared for girls and boys and parents/guardians and key informants comprising of school teachers which is presented in Table 1 Supplementary 1(a), the second is used only for health workers and community leaders, presented in Table 2 Supplementary 1(a).

The guide consists of main themes, main questions followed up by checklist of topics. For interviewing girls and boys and parents/guardians and school teachers' guide, questions were asked to all participants. However, some questions were asked only for parents/guardians and key informants comprising of school teachers indicated in the guide by (*) and some questions were only for girls and boys indicated by (**).

**Table 1 Supplementary S1(a),** Semi-structured interview guide used in the study for girls and boys and parents/guardians and key informants comprising of school teachers.**Main theme**	**Main question**	**Follow-up question**
Cervical cancer	What is the cervix?<br><br>Have you heard about cervical cancer?<br><br>How can we prevent cervical cancer?<br><br>*Do you think your daughter could get cervical cancer anytime in her life?<br><br>Have you ever had a Pap smear?<br>* Do you know what it is for? How often do you have one? From what age?	Where is the cervix located?<br><br>Do you know what is the cause of cervical cancer?<br><br>Have you ever heard about cervical cancer screening?<br>Do you know how cervical cancer is screened?<br>* Do you think you or anybody in your family can get cervical cancer?<br><br>* Have you heard about Pap smear? If not, short explanation is given.
HPV	Have you heard about HPV?<br>How does one get it? How can it be prevented?	What information have you received so far about HPV?<br>What do you know about the association between sexual habits and HPV?

	Do you think you could get HPV?<br><br>* Do you think your daughter or son could get HPV?<br>What effects does it have on males and females?	How is HPV transmitted? Who is at risk of infection? Who can get infected of HPV?<br>* Do you think that your children can get infected during their life?<br>What diseases can HPV cause?
HPV vaccination	What are the benefits of HPV vaccine?<br><br>What consequences (negative) can the vaccination have?<br><br>Who should be vaccinated against HPV?<br>What do you think about the fact that in Kenya HPV-vaccination is not offered to boys?<br><br>* Do you think your daughter needed the HPV vaccine?<br><br>* Has anyone told you that your daughter should get HPV vaccination? Who told you, how?<br><br>* What fears about HPV vaccine have you heard in the community?<br><br>** Have you heard teachers talk about the HPV vaccine?<br>** What have they said?<br>** What do you think they believe about the vaccine?<br>** Have you ever heard a doctor or a nurse talk about the vaccine?<br>** How do you discuss/talk about HPV vaccination among your friends?	What do you know about HPV vaccine?<br><br>Do you have any fears for HPV vaccination?<br><br>Do you think boys can get infected with HPV?<br>What are the most important reasons to vaccinate boys (or not to vaccinate boys)?<br><br>* Who informed you about the HPV vaccine? * Please describe your feelings about the HPV vaccination.<br><br>* Have you ever heard a doctor or a nurse or anybody else talk about the vaccine? * What have they said?<br>* And how did they explain it?<br><br>* What is public opinion? * What are the concerns about HPV vaccination?<br><br>** Who informed you about the HPV vaccine?<br>** What are your thoughts/feelings about the HPV vaccination?<br>** Have you ever heard any information about the HPV vaccine?<br>**What have they said?<br><br>** Do your friends have any concerns about HPV vaccine?
Decision making	* Do you have the necessary information to make a consent decision?<br>What are the reasons for which your daughter was not vaccinated or did not complete the vaccination schedule?<br><br>* In matters related to the health of your daughter (like vaccines and medical procedures) who do you think should decide what to do? Herself? her father? the doctor?	* What information would you need to make a decision regarding vaccination?<br><br>* Would you want to be vaccinated if you were offered HPV vaccination free of charge? What would motivate you to vaccinate your daughter or to complete the vaccination schedule?<br><br>* Do you think that your child can decide on their own if they want to take up vaccines or medical procedures?
Conclusion	Is there anything more you would like to say which I didn't ask you about?	Do you have any remarks for improving compliance with HPV vaccination?

Legend: * questions were asked only for parents/guardians and key informants comprising of school teachers.
** questions were only for girls and boys.

Table 2 Supplementary S1(a), Semi-structured interview guide used in the study to interview guide for health workers and community leaders

Main theme	Main question	Follow-up questions
Cervical cancer	Do you think that cervical cancer is real problem in your community?	How many people have cervical cancer in your community?
HPV	What do you know about prevention of HPV infection?<br>How do you decide when to begin discussing sexual health with patients?	Can HPV infection be prevented?<br><br>HPV vaccination is connected to issues of sexuality. How do you manage discussions related to sexuality with your adolescent patients?
HPV vaccination	What do you think people understand to be the benefits of HPV vaccination?<br><br>What prompts you to discuss/offer vaccination to your patients? How often do they follow your advice for vaccinations?<br><br>How would you describe the initial reaction of the community to the introduction of the HPV vaccination?<br><br>To what extent were the girls and parents/ guardians willing to be participate in the vaccination exercise?<br><br>Have you talked about your experiences with HPV vaccination with your colleagues? What have their experiences been?<br><br>Have you encountered or heard of any common or severe side-effects from HPV vaccination?<br><br>Right now we know boys do not receive the HPV vaccine. What do you think about this?	Is HPV vaccination necessary? Are there any risks with HPV vaccination?<br><br>What has your experience been with offering the HPV vaccine?<br><br>Do you have any concerns about the short-term or long-term safety of the vaccine? Have your colleagues mentioned any concerns? Are there any fears about the HPV vaccine or vaccines in general?<br>Did you have to always coerce the girls and parents/guardians to take the HPV vaccine?<br><br>Do you feel that they are different or similar to yours? Why?<br><br>Have you encountered or heard of any severe reactions?<br><br>Do you think that boys should be vaccinated?
Decision making	Do parents ever seem reluctant to have their daughters vaccinated?<br>[IF YES (and assuming rather frequent “yes” responses)] What have they said? What have their reasons been?<br><br>What about the adolescent girls themselves? How do they feel about getting vaccinated against HPV?	How do parents feel about having their daughters vaccinated against HPV?<br>Tell me examples, why parents or girls are hesitant.<br><br>Do the girls look like they are fearful to receive the vaccine?

	Can you think of any cases where you did not (or would not) offer HPV vaccination to a patient but did (would) offer another vaccine?<br>HPV vaccination entails follow up visits to ensure effectiveness. Are there problems with follow up, or anticipated follow up, ever considerations for you?	Have you encountered incidences where you could not give the HPV vaccine?<br><br>Have you experienced loss to follow up for consequent doses?
Conclusion	Is there anything more you would like to say which I didn't ask you about?	Do you have any ideas for improving compliance with HPV vaccination recommendations?

Supplementary S1(b)

Table 1 Supplementary S1(b), Standards for Reporting Qualitative Research (SRQR) checklist[1]

**Section**	**Line number(s)**
**Title and abstract**	
**Title** - Concise description of the nature and topic of the study Identifying the study as qualitative or indicating the approach (e.g., ethnography, grounded theory) or data collection methods (e.g., interview, focus group) is recommended	Line2-5
**Abstract** - Summary of key elements of the study using the abstract format of the intended publication; typically includes background, purpose, methods, results, andconclusions	Line16-30
**Introduction**	
**Problem formulation - Description and significance of the problem/phenomenon** studied; review of relevant theory and empirical work; problem statement	Line96-110
**Purpose or research questio**n - Purpose of the study and specific objectives orquestions	Line117-121
**Methods**	
**Qualitative approach and research paradigm** - Qualitative approach (e.g., ethnography, grounded theory, case study, phenomenology, narrative research)and guiding theory if appropriate; identifying the research paradigm (e.g., post positivist, constructivist/ interpretivist) is also recommended; rationale**	Line141-153
**Researcher characteristics and reflexivity** - Researchers' characteristics that may influence the research, including personal attributes, qualifications/experience, relationship with participants, assumptions, and/or presuppositions; potential or actual interaction between researchers' characteristics and the research questions,approach, methods, results, and/or transferability	Line174-185
**Context** - Setting/site and salient contextual factors; rationale**	Line124-139
**Sampling strategy** - How and why research participants, documents, or events were selected; criteria for deciding when no further sampling was necessary (e.g., sampling saturation); rationale**	Line145-153
**Ethical issues pertaining to human subjects** - Documentation of approval by anappropriate ethics review board and participant consent, or explanation for lack thereof; other confidentiality and data security issues	Line202-207
**Data collection methods** - Types of data collected; details of data collection procedures including (as appropriate) start and stop dates of data collection andanalysis, iterative process, triangulation of sources/methods, and modification ofprocedures in response to evolving study findings; rationale**	Line162-172<br>Line174-182
**Data collection instruments and technologies** - Description of instruments (e.g.,interview guides, questionnaires) and devices (e.g., audio recorders) used for datacollection; if/how the instrument(s) changed over the course of the study	Line162-172<br>Line175-177
**Units of study** - Number and relevant characteristics of participants, documents, orevents	Line145-153

included in the study; level of participation (could be reported in results)	Line210-215
**Data processing** - Methods for processing data prior to and during analysis, including transcription, data entry, data management and security, verification ofdata integrity, data coding, and anonymization/de-identification of excerpts	Line145-146<br>Line187-192<br>Line166-169
**Data analysis** - Process by which inferences, themes, etc., were identified and developed, including the researchers involved in data analysis; usually references aspecific paradigm or approach; rationale**	Line187-200<br>Line202-207
**Techniques to enhance trustworthiness** - Techniques to enhance trustworthiness and credibility of data analysis (e.g., member checking, audit trail,triangulation); rationale**	Line187-200
**Results/findings**	
**Synthesis and interpretation** - Main findings (e.g., interpretations, inferences, andthemes); might include development of a theory or model, or integration with prior research or theory	Line218-237<br>Line240–302<br>Line305-399<br>Line368-409<br>Figure 1<br>Table1-2
**Links to empirical data** - Evidence (e.g., quotes, field notes, text excerpts,photographs) to substantiate analytic findings	Line261-451<br>Figure 1<br>Table1-2
**Discussion**	
**Integration with prior work, implications, transferability, and contribution(s) tothe field -** Short summary of main findings; explanation of how findings and conclusions connect to, support, elaborate on, or challenge conclusions of earlier scholarship; discussion of scope of application/generalizability; identification of unique contribution(s) to scholarship in a discipline or field	Line454-561<br>Line569-582
**Limitations** - Trustworthiness and limitations of findings	Line563-567
**Other**	
**Conflicts of interest** - Potential sources of influence or perceived influence onstudy conduct and conclusions; how these were managed	Line605-607
**Funding** - Sources of funding and other support; role of funders in data collection, interpretation, and reporting	Line593-595

Legend: [1] Reference: O'Brien, B.C.; Harris, I.B.; Beckman, T.J.; Reed, D.A.; Cook, D.A. Standards for Reporting Qualitative Research: A Synthesis of Recommendations. *Academic Medicine* **2014**, *89*

Supplementary S1(c)

Supplementary S1(c) consists of: 1-Project summary-shared with participants. 2-Informed consents (children-adults).

1-Project summary-shared with participants

Project summary

Title: Perceptions of Human Papilloma Virus Vaccination among school girls/boys, parents/guardians and key informants comprising of school teachers, health workers and community leaders in Kenya: A qualitative study

Study authors: Diana Wangeshi Njuguna and Orsolya Varga

Introduction

Cervical cancer is one of the leading causes of cancer deaths in women globally majority of these occurring in the developing countries. Cancer of the cervix is largely a preventable cancer and evidence has shown that access to screening and early detection is very essential to maximize on the chances of successful treatment of precancerous lesions or suspicion of cervical cancer.

There is high evidence to show that HPV vaccination prevents the incidence of cervical cancer by providing a prophylactic efficacy against cervical pre-cancer in adolescent girls and women who received the HPV vaccination. HPV vaccination contributes to prevention of cervical cancer hence is a public health priority.

The effectiveness and benefits of the HPV vaccine has been demonstrated in several countries as there is a high level of interest to reduce the burden of cervical cancer. Comprehensive HPV vaccination and public education could significantly prevent over half of all cervical cancer deaths.

We are conducting this research work to explore

1. The knowledge and acceptance of HPV vaccination among school girls, boys, parents/guardians and key informants comprising of school teachers, health workers and community leaders.
2. The attitudes and concerns of school girls, boys, parents/guardians and key informants comprising of school teachers, health workers and community leaders regarding HPV vaccines and HPV vaccination.
3. The willingness of the school girls' and their parent's/guardian's to participate in the HPV vaccination program.

To meet these objectives, we wish to conduct explorative qualitative semi-structured interviews.

2-Informed consent form

Consent Form for Children

Research Title:
Perceptions of Human Papilloma Virus Vaccination among school girls, parents/guardians and key informants comprising of school teachers, health workers and community leaders in Kenya: A Qualitative Interview

Researchers' Name: Diana Wangeshi Njuguna, Orsolya Varga

I have read the participation information sheet and have had the opportunity to ask the researcher any further questions I may have had. I understand that my participation in this research is voluntary and I may withdraw at any time from the study without affecting my access to healthcare services in any way.
I understand that the risks to me are minimal in this study and have read the information sheet and asked any questions I may have about the risks. I understand that I will be asked during an interview which takes approximately 30 minutes. The interview will be audio recorded. Following the transcription, the audio file will be destroyed, the text anonymized making impossible identification of participants. Thus, names of participants will not be used.

If I have any concerns or complaints regarding the way, the research is or has been conducted I can contact the University of Eastern Africa, Baraton Ethics Review Committee.
By signing below, I am consenting to participating in the interview. I understand that information from me will be used for the PhD thesis of Diana Wangeshi Njuguna and possibly other published studies and I consent for it to be used in this manner.

I give permission for my child ___________________________________ to participate in this research. (child's name)
Parent/ Guardian Signature _____________________________ Date____________________
Name (please print) ____

Child's signature ____

Consent Form for Adults

Research Title:
Perceptions of Human Papilloma Virus Vaccination among school girls, parents/guardians and key informants comprising of school teachers, health workers and community leaders in Kenya: A Qualitative Interview

Researchers' Name: Diana Wangeshi Njuguna, Orsolya Varga

I have read the participation information sheet and have had the opportunity to ask the researcher any further questions I may have had. I understand that my participation in this research is voluntary and I may withdraw at any time from the study without affecting my access to healthcare services or work in any way.
I understand that the risks to me are minimal in this study and have read the information sheet and asked any questions I may have about the risks. I understand that I will be asked during an interview which takes approximately 30 minutes. The interview will be audio recorded. Following the transcription, the audio file will be destroyed, the text anonymized making impossible identification of participants. Thus, names of participants will not be used.

If I have any concerns or complaints regarding the way, the research is or has been conducted I can contact the University of Eastern Africa, Baraton Ethics Review Committee

By signing below, I am consenting to participating in the interview. I understand that information from me will be used for the PhD thesis of Diana Wangeshi Njuguna and possibly other published studies and I consent for it to be used in this manner.

Name (please print) ____

Caregiver/parents' signature ____

Date___________________
